# Metagenomic Analysis of Therapeutic PYO Phage Cocktails from 1997 to 2014

**DOI:** 10.3390/v9110328

**Published:** 2017-11-03

**Authors:** Julia Villarroel, Mette Voldby Larsen, Mogens Kilstrup, Morten Nielsen

**Affiliations:** 1 Department of Bio and Health Informatics, Technical University of Denmark, Kemitorvet, Building 208, 2800 Kongens Lyngby, Denmark; julvi@bioinformatics.dtu.dk; 2 GoSeqIt ApS, Ved Klædebo 9, 2970 Hørsholm, Denmark; MVL@goseqit.com; 3 Department of Biotechnology and Biomedicine, Technical University of Denmark, Matematiktorvet, Building 301, 2800 Kongens Lyngby, Denmark; mki@bio.dtu.dk; 4 Instituto de Investigaciones Biotecnológicas, Universidad de San Martín, 1650 San Martín, Buenos Aires, Argentina

**Keywords:** PYO phage cocktail, metagenomics, human phage therapy

## Abstract

Phage therapy has regained interest in recent years due to the alarming spread of antibiotic resistance. Whilst phage cocktails are commonly sold in pharmacies in countries such as Georgia and Russia, this is not the case in western countries due to western regulatory agencies requiring a thorough characterization of the drug. Here, DNA sequencing of constituent biological entities constitutes a first step. The pyophage (PYO) cocktail is one of the main commercial products of the Georgian Eliava Institute of Bacteriophage, Microbiology and Virology and is used to cure skin infections. Since its first production in the 1930s, the composition of the cocktail has been periodically modified to add phages effective against emerging pathogenic strains. In this paper, we compared the composition of three PYO cocktails from 1997 (PYO97), 2000 (PYO2000) and 2014 (PYO2014). Based on next generation sequencing, de novo assembly and binning of contigs into draft genomes based on tetranucleotide distance, thirty and twenty-nine phage draft genomes were predicted in PYO97 and PYO2014, respectively. Of these, thirteen and fifteen shared high similarity to known phages. Eleven draft genomes were found to be common in the two cocktails. One of these showed no similarity to publicly available phage genomes. Representatives of phages targeting *E. faecalis*, *E. faecium*, *E. coli*, *Proteus*, *P. aeruginosa* and *S. aureus* were found in both cocktails. Finally, we estimated larger overlap of the PYO2000 cocktail to PYO97 compared to PYO2014. Using next generation sequencing and metagenomics analysis, we were able to characterize and compare the content of PYO cocktails separated by 17 years in time. Even though the cocktail composition is upgraded every six months, we found it to remain relatively stable over the years.

## 1. Introduction

Phage therapy, the use of phages to cure bacterial infections, has received much attention in recent years due to the emergence and rapid spread of antibiotics resistance. In fact, resistance genes towards last resort treatments for multidrug-resistant bacteria are reported to be circulating all around the world. This highlights an urgent need to coordinate a global effort in the search for antibiotics adjuvants or alternative improved treatments [1,2,3].

The practice of phage therapy was reported shortly after phage discovery in 1915 [4], when a sudden enthusiasm emerged towards what was believed to be the cure for almost any disease, even before the biology of phages was fully understood [5,6]. The initial excitement rapidly faded, when phage therapy failed to meet the high expectations, and its practice in western countries soon became obsolete following the discovery of penicillin in 1928 and the advent of the antibiotic era [7].

Despite the displacement of phage therapy by antibiotics in western countries, former Soviet Republics pursued investigations on phages over decades, which today provide a rich trove of knowledge in the field. The related literature has been thoroughly reviewed by Sthephen Abedon et al. [6]. The world leading institution for phage therapy, The Eliava Institute of Bacteriophage, Microbiology and Virology, is located in the former Soviet Republic of Georgia and was founded by the Georgian microbiologist George Eliava in 1923.

In their clinical application, phages are used either as single therapeutic phages, prepared against specific bacterial strains resistant to antibiotics, or phage cocktails which have a broad spectrum of activity towards a set of the most prevalent bacterial strains considered a threat to human health [8]. While the first approach is promoted by the Hirszfeld Institute in Poland, the second is mostly used by the Eliava Institute laboratories, where cocktails’ compositions are updated twice per year by adding new phages to target emerging virulent bacteria [5].

Drug regulatory agencies in western countries, European Medicine Agency and Food and Drug Administration (FDA), are expected to require a comprehensive characterization of the components of a cocktail for it to be considered applicable in healthcare. Whole genome sequencing can be deployed for this purpose, along with methods to predict the host of the draft genomes.

In previous studies, the composition of the intestiphage (INTESTI) cocktail from the Georgian Eliava Institute and the ColiProteus cocktail, which is produced by the Russian company Microgen, have been investigated [9,10]. Among other exciting discoveries, these studies identified a new *Proteus* phage genome sequence. However, both studies only examined the composition of a single batch of cocktail and did not look into changes in the composition of the cocktails over years.

In this metagenomic study, we have sequenced and compared the genomic composition of two pyophage (PYO) cocktails, one from 1997, here referred to as PYO97, and the other from 2014, PYO2014. Upon sequencing the DNA of the cocktails and trimming the reads, we assembled the reads into contigs and further binned the contigs from each sample into phage draft genomes. We then compared these draft genomes to phage sequences previously deposited in public databases and examined which draft genomes were common to both samples and in which abundances. Finally, we predicted the host for each phage draft genome. For a third batch of PYO cocktail from 2000 (PYO2000), we were not able to recover phage draft genomes, but we compared the sequence reads to the draft genomes from PYO97 and PYO2014 and found PYO2000 to resemble PYO97 the most.

## 2. Materials and Methods

**Figure 1:**
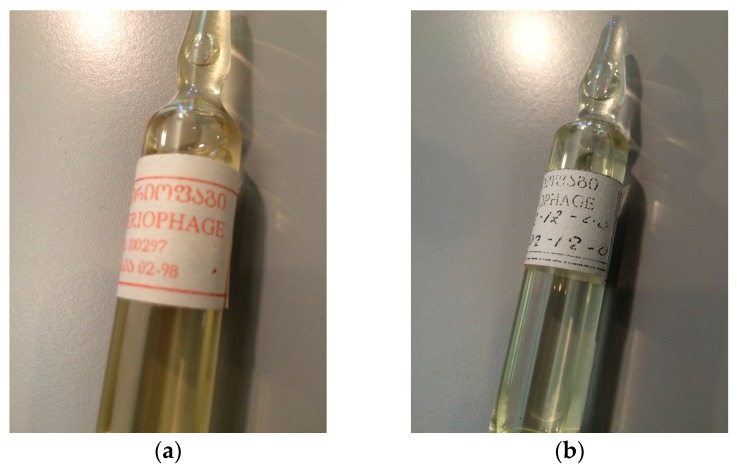

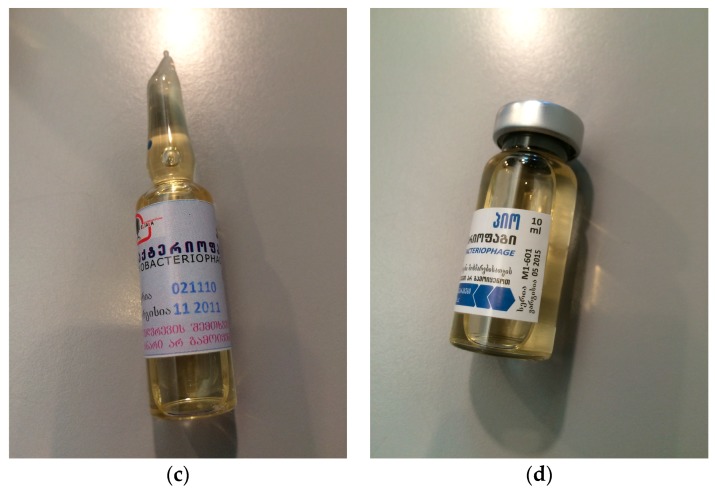
The four batches of pyophage (PYO) cocktail investigated here. The glass ampoules are dated (**a**) 1997; (**b**) 2000; (**c**) 2010 and (**d**) 2014.

Glass vials containing about 10 mL of each of the four PYO phage cocktails—1997, 2000, 2010, and 2014—were kindly provided by Elizabeth Kutter of The Evergreen State College, Olympia, and prepared for sequencing. The bottles are depicted in Figure 1.

### 2.1. DNA Extraction and Library Preparation

The DNA was extracted and isolated using the NORGEN Phage DNA Isolation Kit (Cat. # 46800, Thorold, ON, Canada) following the manual. The extracted DNA was kept at −20 °C until library preparation. PYO97 and PYO2014 had a DNA concentration of 6.06 ng/µL and 1.12 ng/µL, respectively, and a 260/280 ratio within the desired range of 1.8–2.0. PYO2000 and PYO2010 had a DNA concentration of 1.62 ng/µL and 1.61 ng/µL, respectively, but a 260/280 ratio outside the desired 1.8–2.0 range. Due to the 260/280 range, we decided to only process PYO2000 further, and when the resulting sequence reads were of poor quality, refrained from sequencing PYO2010 at all. DNA libraries from PYO97, PYO2000, and PYO2014 were prepared from 1 ng of sample DNA using the NexteraXT Sequencing kit (San Diego, CA, USA) according to the manufacturer’s instructions. The resulting libraries were sequenced using the Illumina MiSeq platform (San Diego, CA, USA) yielding 250 bp long paired-end reads.

### 2.2. Read Trimming

Reads from PYO97, PYO2000, and PYO2014 were checked for quality with Fast Quality Control (FastQC) [11], which produces several different statistics to enable assessment of the quality of short sequence reads. When we, in the following, classified reads as of low or high quality, we based this on the “Per base sequence quality”. Reads were trimmed using Prinseq-lite 0.20.4 [12] with the following settings: -trim_qual_right 20 -min_qual_mean 20 -min_len 35 -trim_left 20 -trim_right 10 -derep 14. Non-parallel reads, resulting from trimming, were compensated using cmpfastq [13].

Reads mapping to PhiX174 phage (NC_001422.1), which is used as an internal control in Illumina sequencing, were removed by running MGmapper [14]. MGmapper is a pipeline that takes a fastq file as input and aligns reads to built-in databases using Burrows-Wheeler Alignment algorithm (BWA) [15]. If none of the databases is specified (option -C 0), the program maps the reads to the PhiX174 genome and returns a fastq file of unmapped reads. MGmapper was launched with the following command: MGmapper_PE.pl -i F.fastq -j R.fastq -R -k -C 0 -S.

The reads quality of PYO2000 was low, even after trimming and removal of PhiX174 reads, therefore this sample was excluded from further analyses until we eventually calculated the distances in composition between the three cocktails; see section *Distances in compositions of the cocktails* in Material and Methods.

### 2.3. Read Mapping

Using Kraken [16], reads from PYO97 and PYO2014 were mapped to the Virus database, which contains complete viral genomes from RefSeq (as of May 2017). Kraken assigns taxonomic labels to metagenomic sequences by searching for exact-matching k-mers (oligonucleotides of length k) between a read and a database of k-mers present in a set of organisms. The Kraken database also stores information about the phylogeny of the organisms. Hence, whenever a query k-mer is present in two or multiple organisms in the database, Kraken assigns the hit to the lowest common ancestor that has these organisms as descendants. Further, reads from PYO97 and PYO2014 were mapped using the Best Mode of MGmapper (option -C) to the built-in databases Bacteria, Archaea, MetaHitAssembly, HumanMicrobiome, Bacteria_draft, Human, Virus, and Fungi downloaded from National Center for Biotechnology Information (NCBI) in June 2017. MGmapper classifies sequences based upon BWA read mapping to a database of reference sequences, allowing for nucleotide variations, inserts and deletions.

### 2.4. Assembly and Contigs Binning

Reads from PYO97 and PYO2014 were assembled into contigs using the metaSPAdes [17] tool from the SPAdes assembly tool kit (version 3.10.1, Saint Petersburg, Russia) [18] with increasing k-mer lengths (21, 33, 55, 77, 99, 127) as suggested in the software manual.

Metagenome Binning with Abundance and Tetra-nucleotide frequencies (MetaBAT) [19], the software used in this study for binning of contigs into draft genomes, requires the assembly in a fasta file and a sorted Binary Alignment Map (BAM) file as input. Reads from PYO97 and PYO2014 were therefore mapped (BWA 0.7.15) [15] to the respective contigs and the resulting BAM files were sorted using SAMtools sort (SAMtools 1.4) [20]. The assembly fasta file and the sorted BAM file were fed to MetaBAT, v0.32.4 for each sample separately. The samples were binned based on tetranucleotide frequency distance probability. We set the minimum contig length to 2000 bp as previously done [9], the minimum bin size to the minimum that MetaBAT allows, which is 10,000 bp, and the bootstrapping to be run 100 times. MetaBAT was ran in *specific* mode: --p1 90 --p2 90 --pB 30 --minProb 80 --minBinned 40 --minCorr 96, to minimize contigs belonging to different phages being binned together.

### 2.5. Finding the Most Similar Reference Genome

Phage whole genome sequences (WGS) were downloaded from the NCBI viral RefSeq database [21] and PhAnToMe [22] resulting in 3889 unique WGS as of May 2017. To find the closest reference to each bin from PYO97 and PYO2014, we ran MetaPhinder [23]. MetaPhinder is a Blast-based method, which for a given query entry provides a measure, the percentage Average Nucleotide Identity (ANI), that integrates multiple hits of the query genome to all sequences in a database. The ANI value is calculated as

(1) $$\%ANI=\frac{\sum_{i=1}^{n}id_{i}*al_{i}}{\sum_{i=1}^{n}al_{i}}*m_{cov}$$

where n is the number of Blastn hits between the query sequence and all sequences in the database with an e-value of 0.05 or smaller, id is the Blastn % identity value between the query and a given database hit, al is the corresponding Blastn alignment length, and $m_{cov}$ is the coverage of the query sequence over all hits. Using this approach, a Blast database was constructed from each bin and next queried with each of the 3889 phage Whole Genome Sequences (WGS). For each bin-database, MetaPhinder reported the ANI for each query WGS, and the query with the highest ANI was selected as the one matching the bin the most.

### 2.6. Checking Consistency within and between Bins

The trimmed reads devoid of PhiX174 of PYO97 and PYO2014 were aligned to the respective contigs using BWA [15]. The coverage, here the number of reads mapping to the contigs times the read length divided by the length of the contigs in bp, was calculated using samtool depth [20]. If high variance of coverage values were observed for the member contigs of a particular bin, the bin was manually split into smaller bins, each only containing contigs with a confined range of coverage values.

Bins that shared the best matching genome among the 3889 WGS had a similar coverage and no overlapping contigs between them were manually merged.

### 2.7. Bin Annotation

To classify if a given bin is a phage or not, we estimated the ANI of each bin from PYO97 and PYO2014 towards the Blastn database of the 3889 phage WGS described earlier. An ANI threshold of 10% was chosen to discriminate between phage and non-phage query bins. For bins containing more than one contig, a weighted ANI average was calculated as

(2) $$\bar{ANI}=\frac{\sum_{i=1}^{n}ANI_{i}*l_{i}}{\sum_{i=1}^{n}l_{i}}$$

where *n* is the number of contigs in the bin and *l* is the length of the member contigs. HostPhinder [24] was used to predict the bacterial host of the draft genomes. HostPhinder predicts the host of a phage genome sequence by searching for overlapping 16-mers between the query and a database of phage genomes with an annotated host. Upon finding the best matching hits in the database, HostPhinder predicts the host to be the most represented host among the top hits. The prediction is associated with a reliability score from 0 to 1. Only scores higher than 0.1 are considered reliable [24]; we therefore only reported results above this threshold.

### 2.8. Similarities between PYO97 and PYO2014.

To estimate the similarity between bins of PYO97 and PYO2014, MetaPhinder was used as follows: A Blast database of contigs from a given bin from one sample was searched with each contig of a bin of the other sample (the query bin). Next, the query bin was assigned a weighted mean ANI calculated from the ANIs and lengths (*l*) of query contigs, Equation (2). For each database bin, the query bin with the highest $\bar{ANI}$ was considered the matching candidate. The reciprocal ANI was calculated using OrthoANI [25], which takes into account only orthologous fragment pairs between the two sequences.

### 2.9. Phage Draft Genome Visualization

Phage draft genomes were visualized using BLAST Ring Image Generator v0.95 (BRIG) [26]. Alternatively, we ran a customized python script to produce xml files from Blast results and used CGView Java Package to visualize them as circular genomes [27].

### 2.10. Bin Classification

Bins were classified into six categories according to high (ANI ≥ 70%) or medium/low (ANI < 70%) resemblance to a reference genome or to a bin in the other sample. Bins that were more than 10% longer than the best matching reference genome and that included overlapping contigs were classified as *collapsed bins*. Bins with ANI < 10% towards phages in public databases were labeled as *special cases*. Bins composed by more than 20 contigs which were shorter than 7000 bp were too fragmented to be considered drafts of genomes and were therefore also designated as special cases. For simplicity, here we will refer to draft genomes to indicate bins that are not special cases. When the term *bin* is used, then all bins including special cases are intended.

### 2.11. Phage Abundances

To further check whether the phages of one cocktail sample were present in the other and with which relative abundance, we mapped the PYO97 reads to the PYO2014 bins and vice versa using BWA. The bin coverage values, calculated here as the number of reads mapping to the bins times the read length and divided by the length of the bins in bp, were obtained using samtools depth [20].

### 2.12. Distances in Compositions of the Cocktails

We ran Mash v1.1.1 [28] to determine the distances in terms of composition between the samples. Trimmed reads devoid of PhiX174 of samples PYO97, PYO2000 and PYO2014 were used.

Mash enables the comparison of metagenomic samples by splitting them into constituent k-mers and reducing the samples into sketches of representative k-mers. From these size-reduced sketches, Mash can rapidly calculate the Jaccard index based on co-occurring k-mers. Based on the Jaccard index, Mash estimates global mutation distances (0 ≤ D ≤ 1) between samples. The results have a strong correlation with the ANI. We chose a k-mer size of 16, a sketch size of 400 and a minimum of 2 copies of k-mers in order for the k-mer to be considered as a candidate for the sample sketch. Mash was launched as follows:

mash sketch -m 2 -k 16 -s 400 -o distance.msh tmp/*.fq

mash dist distance.msh distance.msh > distances.tab

where tmp/*.fq represents the folder containing the fastq files of interleaved reads for the 3 samples.

To get the bootstrap mean and confidence interval of the distances, pair reads of the 3 samples were separately shuffled with resampling 100 times. In each resampling, Mash made sketches of the 3 samples and calculated pairwise distances between the samples. This resulted in one hundred 3 × 3 distance tables from which the mean and mean squared error of each pairwise distance were calculated.

## 3. Results

### 3.1. Reads Statistics

The DNA from each of the four batches of PYO cocktail was extracted. The yield from PYO2010 was very low and we, accordingly, chose not to sequence it. Table 1 reports the number of reads before and after trimming and removal of PhiX174 reads obtained from PYO97, PYO2000, and PYO2014.

PYO2000 was shown to have poor read quality, with a per base sequence quality significantly lower than PYO97 and PYO2014. On account of this, we only attempted to generate phage draft genomes for PYO97, the first time point and PYO2014, the last time point. The trimmed reads devoid of PhiX174 of PYO2000 were mapped to the draft genomes of PYO97 and PYO2014 to examine genomic overlap; see Material and Methods and the section *Phage abundance and bin comparison* in the Results.

### 3.2. Reads Mapping

To get an overview of what was present in the PYO97 and PYO2014 cocktails, reads were initially mapped to the Kraken Virus database.

**Figure 2:**
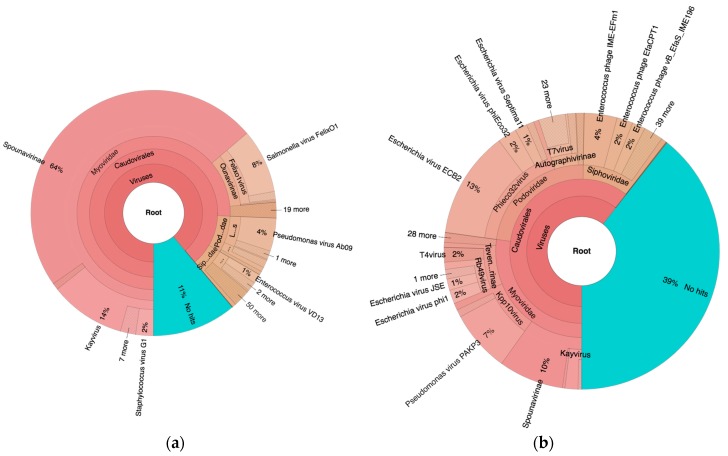
Krona map based on reads from PYO97 (**a**) and PYO2014 (**b**) mapped to the Kraken Virus database. Interactive charts can be found at https://julvi.github.io/PYO97_krona.html and https://julvi.github.io/PYO2014_krona.html for the respective samples.

As seen in Figure 2, 89% and 61% of the reads mapped to viruses of the order *Caudovirales* in PYO97 and PYO2014, respectively. Of these, most mapped to the family *Myoviridae* (85%), while 9% and 6% mapped to *Podoviridae* and *Siphoviridae*, respectively, for PYO97. The ratios of represented phage families within the order *Caudovirales* in PYO2014 were more even: 45% *Myoviridae*, 38% *Podoviridae*, and 17% *Siphoviridae*.

For PYO97, 11% of the reads did not map to any of the sequences in the Kraken Virus database. The corresponding number for PYO2014 was 39%.

To examine if the unmapped reads from above mapped to sequences from other organisms than viruses, MGmapper was next ran using the databases of Bacteria, Archea, MetaHitAssembly [29], HumanMicrobiome [30], Bacteria draft, Human, Viruses and Fungi. No significant mapping to other databases besides Viruses was reported, Table S1.

### 3.3. Assembly and Contigs Binning

In order to detect any draft genome that was common between PYO97 and PYO2014, we proceeded in the assembly and downstream analysis of the two samples with high quality reads.

The assembly yielded 179 and 270 contigs longer than 2000 bp for PYO97 and PYO2014, respectively (Table 2). Note, that while the 270 contigs from PYO2014 in total encompass 2759 kbp to which 6,516,794 reads map, the 179 contigs from PYO97 encompass 3034 kbp to which only 1,924,746 reads map, indicating that the depth of coverage obtained for the PYO97 cocktail is not as high as for the PYO2014 cocktail.

The assembly of metagenome reads often fails to produce entire genomes even for small phage genomes. To arrive at a more complete assembly, MetaBAT was used to group contigs with similar tetranucleotide frequency, allowing to come close to what can be considered draft genomes. MetaBAT produced 33 bins from PYO97 and 31 from PYO2014 and were able to bin more than 90% bp of the contigs longer than 2000 bp for each sample (Table 3).

### 3.4. Consistency within and between Bins

**Figure 3:**
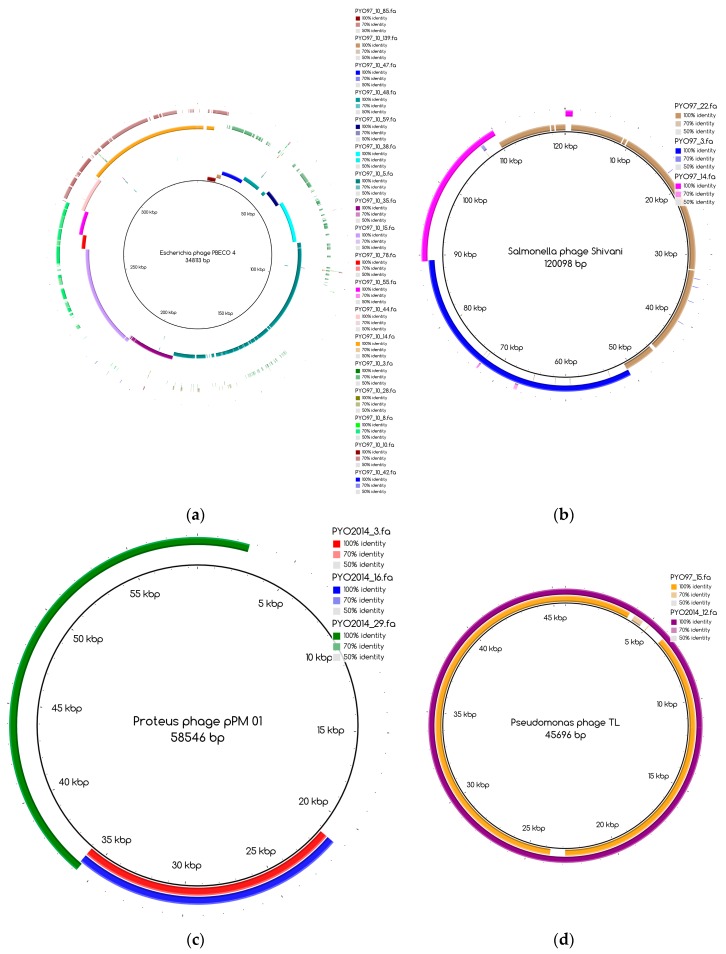

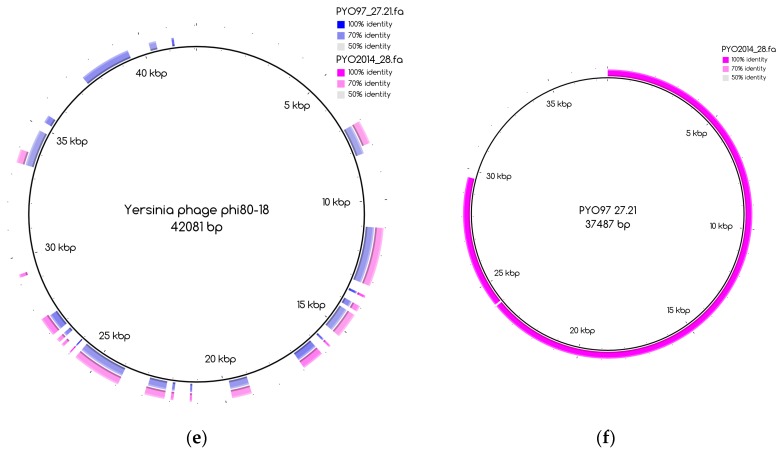
Blast Atlases. (**a**) Example of an original bin, PYO97_10 (coverage 13.9 ± 0.015), that was split into two bins: one made of the contiguous contigs closest to the reference in the middle until the orange contig, bin PYO97_10_85.139.47.48.59.38.5.35.15.78.55.44.14 (coverage 12.4 ± 0.014), and the second one, bin PYO97_10_3.8.10.28.42 (coverage 16.9 ± 0.025), containing the remaining contigs; (**b**) Merging of three bins PYO97_22, PYO97_3 and PYO97_14 into one PYO97_22.3.14 which covers the entire reference genome; (**c**) Collapsed bin in PYO2014; these three contigs have been grouped together to form a collapsed bin. The difference between a normal bin and a collapsed bin is the presence of overlapping contigs in the latter probably derived from shared sequences between species of the same phage family; (**d**) Corresponding draft genomes from the two samples aligning to the reference, *Pseudomonas* phage TL; (**e**) PYO97_27.21 and PYO2014_28 are highly similar and do not resemble any known sequence; (**f**) Alignment of PYO2014_28 to PYO97_27.21.

The bins produced by MetaBAT were composed of between 1 and 50 contigs. In cases where some of the binned contigs overlapped when mapped to the reference sequence, an effort was made to split the bin according to the differences in contig coverage values. After such splits, the newly formed bin generally had a different closest reference genome to the original bin. An illustration of this is shown in Figure 3a. Here, the bin PYO97_10, with *Escherichia* phage PBECO 4 as the closest reference, was split into PYO97_10_85.139.47.48.59.38.5.35.15.78.55.44.14 with the same reference as the original bin and PYO_10_3.8.10.28.42, which in turn had *Escherichia* phage 121Q as the closest reference genome.

Bins mapping to the same reference were merged, if their coverage was in the same range. An example of this is shown in Figure 3b. Here, three bins from PYO97, PYO97_22, PYO97_3 and PYO97_14, which shared a high sequence similarity to *Salmonella* phage Shivani and had coverage values between 58 and 72, were merged into a single bin PYO97_22.3.14 which preserved the reference genome and showed a coverage of 65 with a lower mean standard deviation compared to the original bins. This and other examples of bin merging are listed in Table 4. PYO2014_3.16.29, in our view, represents two or more closely related phages (see Figure 3c), that are identical in the region represented by PYO2014_29, but slightly differ in the regions represented by PYO2014_3 and PYO2014_16.

Eventually, after this manual splitting and merging of bins, 30 and 29 final bins were obtained from PYO97 and PYO2014, respectively.

Two bins of PYO2014 were both composed of 26 contigs, all shorter than 7000 bp. Due to this fragmentation, they were labeled as special cases.

### 3.5. Bin Annotation

We calculated the ANI of the final bins towards the set of publicly available phage genomes to discriminate between phage bins (bins similar to previously sequenced phages) and non-phage bins (bins that share little similarity to known phage sequences). We chose a very stringent threshold of 10% ANI to classify a bin as of phage origin. Using this threshold, five (17%) and three (10%) bins from PYO97 and PYO2014, respectively, were classified as non-phages and added to the special cases. One of the non-phage bins from PYO2014 was already a special case due to its fragmentation, see above. Bins not belonging to the special cases will hereafter be referred to as draft genomes.

To further characterize the draft genomes, we predicted their bacterial hosts using HostPhinder. Table 5 reports the predicted represented bacterial hosts in the two samples.

### 3.6. Similarities between PYO97 and PYO2014

Approximately every 6 months, the Eliava Institute laboratories update the content of the PYO cocktail to cope with the emergence of new clinically problematic bacterial strains. New effective phages are added, while phages added in previous batches slowly dilute, leading to an overall change of the cocktail composition.

We investigated how much overlap in the compositions of PYO97 and PYO2014 was appreciable by looking for common phage draft genomes between the two cocktails. The corresponding pairs of draft genomes between the two samples were determined using MetaPhinder in a pairwise manner as described in Materials and Methods.

Table 6 reports the pairs identified by MetaPhinder, where at least one of the ANI, calculated either by using PYO97’s or PYO2014’s phage drafts as databases was higher than 70%.

The combined results of HostPhinder and pairwise MetaPhinder displayed in Table 6 strongly suggest that the same phages against *E. faecalis* (2), *E. faecium* (1), *E.coli* (2), *P. mirabilis* (1), *P. aeruginosa* (2), and *S. aureus* (1) are present in both samples; where the numbers in parenthesis are the counts of likely identical phages found in both samples which are capable of infecting the specified host.

### 3.7. Draft Genomes Classification

According to their similarity to reference genomes and to the presence of a likely counterpart at the other time point (see Materials and Methods), draft genomes were classified within the categories listed in Table 7. The special cases include highly fragmented bins and non-phage bins. For these reasons, special cases are referred to as bins and not as draft genomes. Table 7 also displays the number of draft genomes/bins from each sample belonging to each category. As an illustrative example, the six draft genomes from PYO97 in category 1, have high similarity to a reference genome and to draft genomes in PYO2014. One example of pairs of corresponding draft genomes is given by PYO97_15 and PYO2014_12, Figure 3d. The two draft genomes share high similarity to Pseudomonas phage TL.

**Figure A1:**
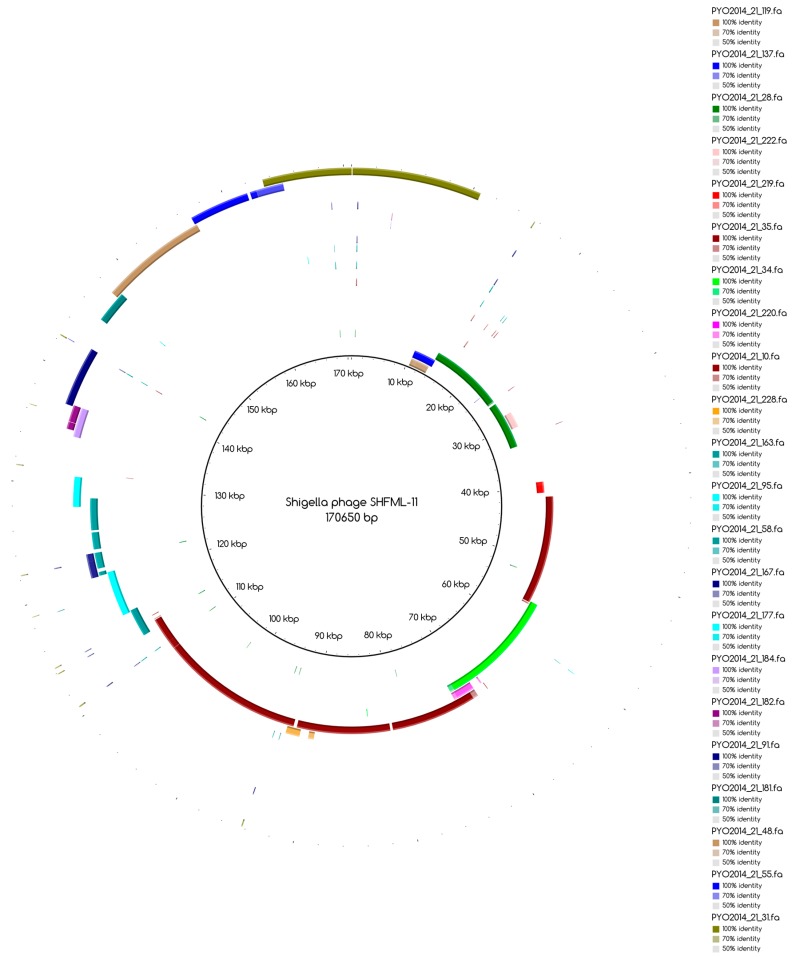
Blast Atlas of PYO2014_21 towards the reference *Shigella* phage SHFML-11.

The number of draft genomes belonging to each category does not necessarily match between the two samples, even for the categories of draft genomes with a counterpart in the other sample, categories 1 and 3. This is, for instance, the case for draft genome PYO97_29, category 1, mapping to the collapsed draft genome PYO2014_21, which belongs to the fifth category, Figure A1. Table 8 and Table 9 provide a general overview of the phage draft genomes found in PYO97 and PYO2014, respectively, together with an indication of the most likely taxonomic group they belong to. For a more thorough description of the draft genomes in each category, see Tables S2 and S3 for PYO97 and PYO2014, respectively. A case worth noticing is that of the draft genomes PYO97_27.21 and PYO2014_28 in category 3. These draft genomes share similarity with ANI > 70%, but have low ANI to the common reference genome, *Yersinia* phage phi80-18 (refer to, Figure 3e,f for an illustration of the overlap between the two bins). This could suggest that the PYO97_27.21 and PYO2014_28 draft genomes represent a previously uncharacterized phage.

It is worth noticing that the percentage of reads that align to the bins with ANI < 40 towards known sequences was 6.87% and 22.79% for PYO97 and PYO2014, respectively. These percentages align with the differences in percentages of unclassified reads between the two samples, as found when using Kraken in paragraph 3.2: 11% for PYO97 and 39% for PYO2014. However, the results from BWA and Kraken analyses are not directly comparable since BWA alignment allows for indels and point mutation [15], while Kraken only reports exact matching k-mers [16].

### 3.8. Phage Abundances and Bin Comparison

To estimate the relative abundances of bins in PYO97 and PYO2014, we calculated the bin coverage of the PYO97’s and PYO2014’s bins by the reads of the samples PYO97 and PYO2014. To account for the difference in the number of reads between sample PYO97 and PYO2014, we normalized the coverage values by the total number of reads of the respective sample.

**Figure 4:**
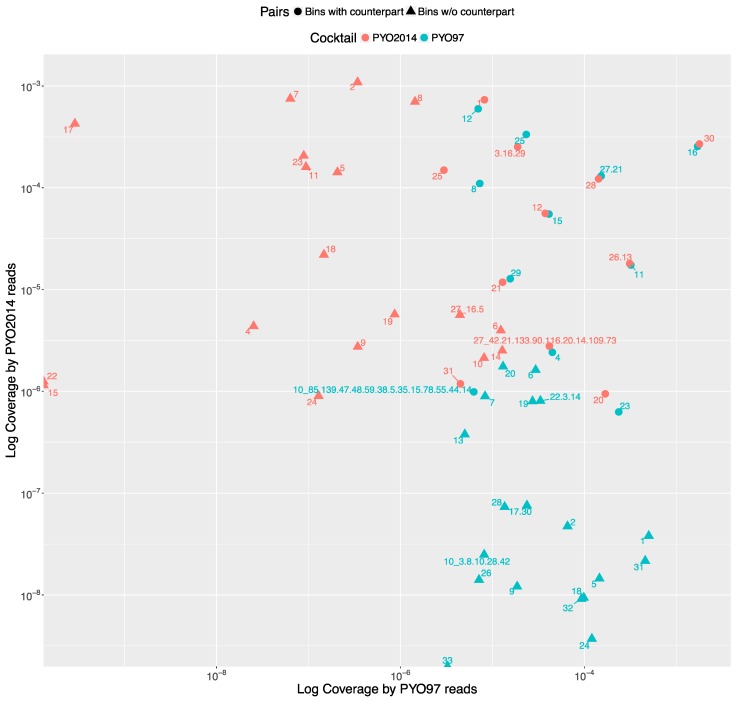
All the bins from PYO97 and PYO2014 are plotted according to the Log to base 10 of the coverage by PYO97’s reads, x-axis and PYO2014’s reads, y-axis. Bins from PYO97 are depicted in blue, whilst bins from PYO2014 are red. Circle shaped data points represent pairing bins between the two samples, i.e., bins for which MetaPhinder found a matching counterpart in the other sample with a ANI > 70%; see Table 6 and Material and Methods, *Estimate similarities between PYO97 and PYO2014*.

The distribution of the bins according to the bin coverage by the reads of PYO97 and PYO2014 is shown in Figure 4. Circles represent draft genomes listed in Table 6 having a counterpart in the other sample. These draft genomes had generally high abundances in both samples, which is deducible from the position of circle data points in the top right corner of the graph. PYO97_27.21 and PYO2014_28 offer an interesting example, as these two draft genomes are almost completely overlapping in terms of relative abundance in the two samples. As stated earlier, these two draft genomes have high ANI and both had low similarity to the common best reference, *Yersinia* phage phi80-18. HostPhinder predicted *Yersinia enterocolitica* to be the host of both, yet with a low confidence, see last column in Tables S1 and S2. Figure 3f displays the sequence similarity between the two bins. These results thus further support the conclusion that this phage draft is an example of a previously unsequenced phage genome.

The bottom right corner of Figure 4 is populated by PYO97’s bins with low bin coverage by PYO2014’s reads, whilst the top left clusters PYO2014’s bins with low bin coverage by PYO97’s reads. The bins in these two parts of the figure are thus most likely phages added (top left corner) or removed (lower right corner) when constructing the cocktails at the two time points: 1997 and 2014.

We next determined the distances in composition between samples PYO97, PYO2000 and PYO2014 using Mash. The algorithm searches shared k-mers between samples and gave a measure of global mutation distance that takes continuous values between 0 and 1. For each representative k-mer, Mash does not take into account how many of those k-mers are present in each sample, only whether it is present or not. Therefore, the distances are to be considered qualitatively as distances in the variety of phages between samples and not as differences in phage abundances.

Distances are, in general, low between the samples (D < 0.2), Table 10, as expected since the different samples are of the same cocktail and contain mostly shared sequences. PYO2014 has the highest distance to the other two samples. From this, it can be derived that a higher number of phages are unique to PYO2014 and absent in the other samples. Conceivably from the date of production, PYO97 and PYO2000 are less distant to each other (0.113 ± 0.0006) than they are to PYO2014, (0.132 ± 0.0008 and 0.138 ± 0.0009, respectively).

## 4. Discussion

In this paper, we aimed to investigate the composition of four batches of PYO cocktail, produced at the Eliava Institute in 1997, 2000, 2010, and 2014, by means of sequencing and metagenomic analysis. The PYO cocktails from 1997 and 2014 had been stored in a fridge at approximately 4 °C. We were able to extract DNA of high quality from these samples and likewise obtained high-quality sequence reads. We did not test the infectivity of the phages in the cocktails, but have previously found that phages from another cocktail from the Eliava Institute, the INTESTI cocktail, retain their infectivity after storage under similar conditions for at least two years [9]. The phages in the INTESTI cocktail lost their infectivity when they were frozen by mistake without the addition of glycerol. Similarly, the PYO cocktails from 2000 and 2010 had been frozen without the recommended addition of glycerol [31]. Following thawing, we were not able to extract enough DNA of good quality from these cocktails and only obtained sequence reads from PYO2000, which were furthermore of a poorer quality than from PYO97 and PYO2014. We did not test whether the phages in PYO2000 and PYO2010 had also lost their infectivity, but expect that they had. It is worth mentioning that the recommended long-term storage of phages is freezing −80 °C after addition of glycerol [31]. Alternatively, phages can be freeze dried and stored at room temperature [32].

The reads from PYO97 and PYO2014 were assembled into contigs, which were binned into phage draft genomes in a reference independent manner. This is contrary to what was previously done for the INTESTI cocktail [9], where contigs were binned based on Blast searches to public databases. For the purpose of binning the contigs, we used MetaBAT, a method that bins according to the tetranucleotide frequency distances of the contigs. Further, MetaBAT is able to use the co-abundances of contigs in multiple samples, i.e., the consistency in coverage fluctuations of groups of contigs between samples. The method is optimized to handle huge assemblies for a number of samples greater than ten. Since our study involved only two samples of good quality, MetaBAT could not base contigs binning on the co-abundance information, but only on the tetranucleotide frequency distances. This might explain why, consequent to binning, we had to manually curate the generated bins. Two phage draft genomes, one from each sample, were in fact each manually split into two phage draft genomes and other bins were merged according to the coverage consistency and closest reference genome, resulting in five merged draft genomes.

Phage draft genomes were further classified into categories based on their similarity to a reference genome and/or to a phage draft genome in the other sample. This allowed us to identify a group of phage draft genomes that were highly similar to a reference genome and present in both samples. These included draft genomes predicted to target *E. faecalis*, *E. faecium*, *E. coli*, *P. mirabilis*, *P. aeruginosa*, and *S. aureus*. Other near-complete and partial draft genomes, even if without a counterpart in the other sample or reference genome, were predicted to target also *C. sakazakii*, *K. pneumoniae*, *Shigella,* and species of *Salmonella*. Only the prediction of phages targeting *C. sakazakii* and *K. pneumoniae* were counter to our expectations as the declared activity of the PYO cocktail includes *Shigella*, *Salmonella*, *E. coli*, *Proteus*, *S. aureus*, *P. aeruginosa* and *Enterococcus*. Previous studies have shown the close taxonomic relatedness between bacteria of the Enterobacteriaceae family [33,34], which includes *Escherichia*, *Klebsiella*, *Salmonella* and *Shigella*, suggesting that the prediction of *K. pneumoniae* might be a misprediction. Besides, even though phages are usually strain-specific, phages capable of infecting distinct but related hosts, polyvalent phages, are commonly observed among phages of Enterobacteria [35,36,37,38], which does not rule out the presence of this type of phages in the cocktail.

To the best of our knowledge, the ANI thresholds for when a phage belongs to a certain species, genus, or family have not been defined. However, we suggest that the phage draft genomes in category 1 and 2 represent phages that likely belong to previously sequenced phage species or at least previously defined genera. Examples include PYO97_11 and PYO2014_26.13 that both closely resemble *Pseudomonas* phage PEV2, a N4likevirus. The phage draft genomes in categories 3 and 4 are, on the other hand, likely to be the first representatives of previously undefined genera, in some cases perhaps even previously undefined sub-families or families, with ANI to the closest reference genomes from 10% to 70%. Examples include PYO97_27.21 which closely resembles PYO2014_28. Both phage draft genomes have an ANI to the closest reference of only approximately 20%. Another example is PYO2014_7, which does not have a counterpart in PYO97 and only has a ANI of 14.3% to the closest reference.

A total of twenty-two new near-complete or partial draft genomes were discovered, which did not resemble any publicly available genomes, or had only poor similarity to one. One of these phage draft genomes was even found to be present in both samples and with high relative abundance (PYO97_27.21/PYO2014_28).

In correspondence to this high number of previously unsequenced phage draft genomes, we also observed a relatively high percentage of reads that could not be mapped to any known phage genome. For PYO97, 11% of the reads could not be mapped to known phage sequences, while the corresponding percentage for PYO2014 was 39%. This relates to the continued scarceness of phage genome representation in public databases compared to bacterial sequences [39]. A previous study from 2013 was able to map 61% of the reads from the Microgen ColiProteus cocktail to public genomes [10].

For PYO97, 17% of the bins were not predicted to be of phage origin, while for PYO2014 the corresponding percentage was 10. When predicting if bins were of phage origin, we used MetaPhinder with a very stringent threshold of 10% ANI. This is a far more conservative threshold than suggested in the original paper describing the MetaPhinder method [23], where the ANI threshold to classify a contig as of phage origin was set to 1.7% ANI. Further, the performance of MetaPhinder is dependent on the size and diversity of a reference database of previously sequenced phages. We thus consider it likely that the bins predicted to be of non-phage origin are due to a limited diversity in the previously sequenced phage genomes rather than, e.g., contamination. This hypothesis is supported by the analysis using MGmapper, which showed that only a negligible amount of the raw sequence reads mapped to reference databases containing sequences from Bacteria, Archaea, MetaHitAssembly, HumanMicrobiome, Bacteria_draft, Human, Virus, or Fungi. Most of the bins predicted to be of non-phage origin had the highest similarity to sequences annotated as uncultured Mediterranean phages. It is worth noticing that phages annotated as uncultured Mediterranean phages counted 28.8% of the 3889 WGS used to search for references to the bins, which raises the chance that they were randomly selected.

A coverage analysis that included PYO2000 showed a closer similarity of this cocktail batch to the batch from 1997 than that from 2014, in terms of composition. This is also to be expected as there are only 3 years between the production of the first two cocktails compared to the second and the last batches, which were produced with 14 years in between. The phage draft genomes of the PYO97 and PYO2014 cocktails showed huge differences in depth coverages within the samples, indicating as much as a thousand-fold difference between the most and least abundant phages. We speculate that the draft genomes represented by few sequence reads may derive from phages of older batches that have been diluted over time. Alternatively, they may derive from activated prophages integrated in the bacterial hosts used for phage enrichment, as previously suggested [10]. In the previous study by our group of the INTESTI cocktail [9], we did not observe such high differences in abundances. This might, however, be due to the general much lower sequencing depth of the INTESTI cocktail, which would not have allowed for the detection of the phages found at very low concentrations. It is worth pointing out the composition comparison presented here could not account for potential compositional variations within the batches nor for any biases that might have been introduced during sample processing. This is an insight that could be gained by analyzing multiple samples per batch and/or introducing replicates; however, this was beyond the scope of this study.

One of the limitations of the analysis applied here is that neither the lab sample preparation nor the sequencing library construction enriched for RNA sequences. Therefore, likely present *Pseudomonas* phages of potential clinical importance as antimicrobials [40], could not be detected. Besides small RNA coliphages, ssDNA phages were likely missed. In fact, the amplification step of the Illumina sequencing used here is based on the ligation of dsDNA adapters to sheared DNA. Since the ligation occurs between dsDNA fragments, ssDNA phages of the families *Microviridae* and *Inoviridae* could not be efficiently recovered by this approach [41,42]. Furthermore, the binning method that we chose yielded only bins of 10,000 bp or larger. Although we were able to bin more than 90% of the basepairs represented in the contigs, the threshold of 10,000 bp might have sorted out small DNA phages, for instance small *E.coli* phages [43].

## 5. Conclusions

In the present study, we have performed metagenomic sequencing and analysis of phage cocktails produced over 18 years. Some of the observed phages are common to the phage cocktails and are likely to belong to previously defined phage species and genera. However, we also discovered new phages that only poorly resemble any of the whole genome phage sequences found in public databases. They are likely to represent new genera or even new phage families. For a fuller characterization of the content of the cocktails, methods that also allow for RNA isolation and enrichment and binning processes that allow for the formation of smaller bins, is needed. The raw reads from this study are publicly available at http://www.ebi.ac.uk/ena/data/view/PRJEB23244. The draft genomes have been deposited on the European Nucleotide Archive with accession numbers from ERS1989512 to ERS1989570. It is the authors’ hope that this will allow other researchers to continue analyzing and characterizing these phages. The characterization of the cocktail is a first step towards recognizing the PYO cocktail as a regulated drug in western countries.

## Figures and Tables

**Table 1:** Reads statistics.

Sample	^#^ Reads	^#^ Reads after Trimming	^#^ Reads after Removing PhiX174
PYO97	5,228,884 (1,280,000 kbp)	2,035,496 (420,000 kbp)	1,965,233 (410,000 kbp)
PYO2000	1,648,430 (410,000 kbp)	1,366,749 (300,000 kbp)	1,110,522 (240,000 kbp)
PYO2014	18,240,556 (4,470,000 kbp)	6,660,081 (1,380,000 kbp)	6,577,613 (1,370,000 kbp)

^#^ Means “number of”.

**Table 2:** Summary of the assembly results.

Sample	^#^ Contigs	Longest and Shortest Contig	^#^ Contigs Longer than 2 kbp–Percentage of Reads Mapping to the Contigs
PYO97	3005 (4403 kbp)	169,029 bp (128 bp)	179 (3034 kbp)–97.9%
PYO2014	4165 (4865 kbp)	282,352 bp (128 bp)	270 (2759 kbp)–99.1%

^#^ Means “number of”.

**Table 3:** Number of bins yielded by MetaBAT and number and percentage of binned bp out of the total number of bp in contigs larger than 2000 bp.

Sample	^#^ Bins	^#^ Binned bp (Percentage of Binned bp)
PYO97	33	2,735,811 (90.16%)
PYO2014	31	2,494,104 (90.39%)

^#^ Means “number of”.

**Table 4:** The bins to be merged are indicated in the first three columns. The fourth and fifth columns show the resulting merged bin and the closest reference, respectively. Coverage values are in parentheses.

Bin to be Merged 1	Bin to be Merged 2	Bin to be Merged 3	Merged Bin	Shared Reference
PYO97_17 (44.67 ± 0.05)	PYO97_30 (53.82 ± 0.07)		PYO97_17.30 (46.65 ± 0.04)	*Salmonella* phage SSE-121 (NC_027351.1)
PYO97_3 (57.92 ± 0.08)	PYO97_14 (66.42 ± 0.14)	PYO97_22 (71.55 ± 0.14)	PYO97_22.3.14 (65.41 ± 0.08)	*Salmonella* phage Shivani (NC_028754.1)
PYO2014_3 (2294.3 ± 3.8)	PYO2014_16 (2294.98 ± 3.81)	PYO2014_29 (2222.82 ± 2.54)	PYO2014_3.16.29 (1659.65 ± 2.65)	*Proteus* phage pPM_01 (NC_028812.1)
PYO2014_13 (109.82 ± 0.21)	PYO2014_26 (120.43 ± 0.16)		PYO2014_26.13 (118.21 ± 0.14)	*Pseudomonas* phage PEV2 (NC_031063.1)

**Table 5:** Number of phage draft genomes from the two samples which HostPhinder predicted to infect the respective host. Only results having a score higher than the reliability threshold of 0.1 are reported.

Bacterial Host	^#^ Representative Phage Draft Genomes in PYO97	^#^ Representative Phage Draft Genomes in PYO2014
*Enterococcus faecalis*	2	3
*Enterococcus faecium*	1	1
*Escherichia coli*	4	7
*Klebsiella pneumoniae*	1	0
*Proteus mirabilis*	2	1
*Pseudomonas aeruginosa*	3	4
*Salmonella enterica*	2	0
*Salmonella enteritidis*	2	0
*Shigella sonnei*	0	1
*Staphylococcus aureus*	1	1

^#^ Means “number of”.

**Table 6:** Overview of correspondent draft genomes between PYO97 and PYO2014 and the reciprocal ANI. The last column displays the targeted host as predicted by HostPhinder. Bins 10_85…, and 27_42… in the table correspond to bin 10_85.139.47.48.59.38.5.35.15.78.55.44.14 and 27_42.21.133.90.116.20.14.109.73, respectively.

Bin from PYO97	Bin from PYO2014	Reciprocal ANI (%)	Predicted Targeted Bacterial Host
16	30	99.9	*S. aureus*
27.21	28	98.6	*Yersinia enterocolitica* *
11	26.13	97.2	*P. aeruginosa*
12	1	98.8	*E. faecium*
25	3.16.29	99.5	*Proteus mirabilis*
15	12	98.7	*P. aeruginosa*
29	21	96.2	*E. coli*/*Shigella sonnei* **
4	27_42…	98.1	*E. faecalis*
8	25	88.4	*E. coli*
10_85…	31	89.6	*E. coli*
23	20	85.4	*E. faecalis*

* Indicates that the prediction by HostPhinder had a low score and was hence unreliable. ** In this case, HostPhinder predicted a different host for each draft genome.

**Table 7:** Count of draft genomes/bins belonging to each category.

Class	PYO97	PYO2014
(1) Near-complete draft genome with high resemblance to reference phage and counterpart in the other sample.	6	4
(2) Near-complete draft genome with high resemblance to reference phage, but no counterpart in the other sample.	5	8
(3) Partial draft genome with low/medium resemblance to reference phage and counterpart in the other sample.	1	1
(4) Partial draft genome with no resemblance to reference phage and no counterpart in the other sample.	11	8
(5) Collapsed bins.	2	4
(6) Special cases, including highly fragmented bins and bins classified as non-phages.	5	4

**Table 8:** Overview of the phage draft genomes and bins of PYO97 indicating the most likely taxonomic group they belong to. PYO97_10_85… and PYO97_10_3… correspond to PYO97_10_85.139.47.48.59.38.5.35.15.78.55.44.14 and PYO97_10_3.8.10.28.42, respectively.

Bin Name	^#^ Contigs	Size (bp)	Closest Relative in the Database	ANI (%)	Most Likely Taxonomic Group
PYO97					
PYO97 near-complete draft genomes with high resemblance to reference phage and counterpart in PYO2014. (Category 1)					
PYO97_4	1	149,561	*Enterococcus* phage EFDG1 (NC_029009.1)	89.77	*Caudovirales; Myoviridae; unclassified Myoviridae*
PYO97_10_85…	13	344,749	*Escherichi*a phage PBECO 4 (NC_027364.1)	90.848	*Caudovirales; Myoviridae; unclassified Myoviridae*
PYO97_11	1	72,136	*Pseudomonas* phage PEV2 (NC_031063.1)	97.37	*Caudovirales; Podoviridae; N4likevirus; unclassified N4likevirus*
PYO97_15	1	44,667	*Pseudomonas* phage TL (NC_023583.1)	92.02	*Caudovirales; Podoviridae; Luz24virus; Pseudomonas virus TL*
PYO97_16	1	130,932	*Staphylococcus* phage Sb-1 (HQ163896.1)	96.86	*Caudovirales; Myoviridae; Spounavirinae; Spo1virus; unclassified SPO1-like viruses*
PYO97_29	1	169,029	*Shigella* phage SHFML-11 (NC_030953.1)	89.959	*Caudovirales; Myoviridae; Tevenvirinae; T4virus; unclassified T4virus*
PYO97 near-complete draft genomes with high resemblance to reference phage, but no counterpart in PYO2014. (Category 2)					
PYO97_7	7	166,126	*Klebsiella* phage vB KpnM KpV477 (NC_031087.1)	88.66	*Caudovirales; Myoviridae*
PYO97_8	1	38,419	*Enterobacteria* phage 285P (NC_015249.1)	79.568	*Caudovirales; Podoviridae; Autographivirinae; T7virus; unclassified T7-like viruses*
PYO97_22.3.14	3	109,428	*Salmonella* phage Shivani (NC_028754.1)	95.33	*Caudovirales; Siphoviridae; T5virus; Salmonella virus Shivani*
PYO97_24	1	44,541	*Proteus* phage PM 85 (NC_027379.1)	92.726	*Caudovirales; Podoviridae; unclassified Podoviridae*
PYO97_32	3	47,235	*Salmonella* phage vB SenS-Ent1 (HE775250.1)	86.967	unclassified
PYO97 partial draft genome with low/medium resemblance to reference phage and counterpart in PYO2014. (Category 3)					
PYO97_27.21	2	37,487	*Yersinia* phage phi80-18 (NC_019911.1)	22.104	*Caudovirales; Podoviridae*
PYO97 partial draft genomes with no resemblance to reference phage and no counterpart in PYO2014. (Category 4)					
PYO97_1	1	11,445	*Escherichia* phage vB EcoM AYO145A (NC_028825.1)	10.99	*Caudovirales; Myoviridae*
PYO97_5	3	29,155	*Pseudomonas* phage vB Pae-TbilisiM32 (JQ307386.1)	68.72	*Caudovirales; Podoviridae; Autographivirinae*
PYO97_9	1	10,727	*Salmonella* phage BP63 (NC_031250.1)	19.779	*Caudovirales; unclassified Caudovirales*
PYO97_10_3…	5	343,801	*Escherichia* phage 121Q (NC_025447.1)	28.408	*Caudovirales; Myoviridae*
PYO97_13	1	37,843	*Hamiltonella* virus APSE1 (NC_000935.1)	9.777	*Caudovirales; Podoviridae*
PYO97_17.30	7	90,209	*Salmonella* phage SSE121 (NC_027351.1)	58.832	*Caudovirales; Myoviridae; Vequintavirinae*
PYO97_20	1	90,712	*Cronobacter* phage vB CsaP GAP52 (NC_019402.1)	19.54	*Caudovirales; Podoviridae*
PYO97_25	1	25,293	*Proteus* phage pPM_01 (NC_028812.1)	41.01	*Caudovirales; Siphoviridae; unclassified Siphoviridae*
PYO97_26	5	171,908	Cronobacter phage S13 (NC_028773.1)	45.28	*Caudovirales; Myoviridae; unclassified Myoviridae*
PYO97_28	5	30,952	*Salmonella* phage 21 (NC_029050.1)	21.43	*Caudovirales; Myoviridae*
PYO97_31 *	3	69,885	*Salmonella* phage *Felix* 01 (NC_005282.1)	75.359	*Caudovirales; Myoviridae; Ounavirinae*
PYO97 collapsed bins. (Category 5)					
PYO97_12	5	55,452	*Enterococcus* phage IME-EFm5 (NC_028826.1)	69.288	*Caudovirales; Siphoviridae; unclassified Siphoviridae*
PYO97_23	5	73,434	*Enterococcus* phage VD13 (NC_024212.1)	74.273	*Caudovirales; Siphoviridae; Sap6virus*
PYO97 special cases, including bins classified as non-phages. (Category 6)					
PYO97_2	1	11,313	uncultured Mediterranean phage uvMED-GF-C25-MedDCM-OCT-S33-C258 (AP014078.1)	0.704	unknown
PYO97_6	8	23,397	uncultured Mediterranean phage uvMED-CGF-C14B-MedDCM-OCT-S36-C258 (AP013800.1)	1.426	unknown
PYO97_18	1	11,354	*Pseudomonas* phage PRR1 (NC_008294.1)	0.984	unknown
PYO97_19	10	284,533	*Staphylococcu*s phage Sb-1 (HQ163896.1)	18.88	*Caudovirales; Myoviridae*
PYO97_33	3	10,088	uncultured Mediterranean phage uvMED-CGF-C23-MedDCM-OCT-S24-C232 (AP013582.1)	1.131	unknown

* PYO97_31 is 20 kbp shorter than the reference, therefore it was placed in this category, despite the high ANI of the reference genome. ^#^ Means “number of”. The 5th column reports the ANI of the reference genome towards the bin.

**Table 9:** Overview of the phage draft genomes and bins of the cocktail PYO2014, indicating the most likely taxonomic group they belong to. PYO2014_27_42… corresponds to PYO2014_27_42.21.133.90.116.20.14.109.73.

Bin Name	^#^ Contigs	Size (bp)	Closest Relative in the Database	ANI (%)	Most Likely Taxonomic Group
PYO2014					
PYO2014 near-complete draft genomes with high resemblance to reference phage and counterpart in PYO97. (Category 1)					
PYO2014_1	1	42,721	*Enterococcus* phage IME-EFm5 (NC_028826.1)	70.16	*Caudovirales; Siphoviridae; unclassified Siphoviridae*
PYO2014_12	1	47,209	*Pseudomonas* phage TL (NC_023583.1)	97.91	*Caudovirales; Podoviridae; Luz24virus; Pseudomonas virus TL*
PYO2014_27_42…	9	138,228	*Enterococcus* phage EFDG1 (NC_029009.1)	81.548	*Caudovirales; Myoviridae; unclassified Myoviridae*
PYO2014_30	1	138,269	*Staphylococcus* phage ISP (FR852584.1)	99.36	*Caudovirales; Myoviridae; Spounavirinae; Kayvirus; Staphylococcus virus G1*
PYO2014 near-complete draft genomes with high resemblance to reference phage, but no counterpart in PYO97. (Category 2)					
PYO2014_2	1	76,529	*Escherichia* phage ECBP2 (NC_018859.1)	77.91	*Caudovirales; Podoviridae; Phieco32virus; Escherichia virus ECB2*
PYO2014_4	1	282,352	*Pseudomonas* phage phiKZ. (NC_004629.1)	94.53	*Caudovirales; Myoviridae; Phikzvirus*
PYO2014_8	1	36,807	*Enterococcus* phage EFAP-1 (NC_012419.1)	74.45	*Caudovirales; Siphoviridae; unclassified Siphoviridae*
PYO2014_17	1	88,099	*Pseudomonas* phage CHA P1 (NC_022974.1)	94.91	*Caudovirales; Myoviridae*
PYO2014_18	1	147,760	*Enterobacteria* phage phi92 (NC_023693.1)	91.5	*Caudovirales; Myoviridae; unclassified Myoviridae*
PYO2014_23	1	38,847	*Enterobacteria* phage K1F (NC_007456.1)	82.27	*Caudovirales; Podoviridae; Autographivirinae; T7virus; unclassified T7-like viruses*
PYO2014_26.13	2	65,818	*Pseudomonas* phage PEV2 (NC_031063.1)	90.705	*Caudovirales; Podoviridae; Lit1virus; Pseudomonas virus Ab09*
PYO2014_27_16.5	2	139,828	*Enterococcus* phage EFLK1 (NC_029026.1)	90.812	*Caudovirales; Myoviridae; unclassified Myoviridae*
PYO2014 partial draft genome with low/medium resemblance to reference phage and counterpart in PYO97. (Category 3)					
PYO2014_28	1	33,115	*Yersinia* phage phi80-18 (NC_019911.1)	16.79	*Caudovirales; Podoviridae; Autographivirinae*
PYO2014 partial draft genomes with no resemblance to reference phage and no counterpart in PYO97. (Category 4)					
PYO2014_7	1	103,078	*Escherichia* phage bV EcoS AKFV33 (HQ665011.1)	14.32	*Caudovirales; Siphoviridae*
PYO2014_9	1	37,468	*Enterococcus* phage vB IME197 (NC_028671.1)	15.18	*Caudovirales; Siphoviridae*
PYO2014_19	1	43,272	*Pseudomonas* phage vB PaeP Tr60 Ab31 (NC_023575.1)	45.35	*unclassified dsDNA phage*
PYO2014_10	1	10,736	*Escherichia* phage PBECO 4 (NC_027364.1)	3.06	*Caudovirales; Myoviridae*
PYO2014_11	1	13,190	*Escherichia* phage PE3-1 (NC_024379.1)	29.52	*Caudovirales; Podoviridae; Autographivirinae*
PYO2014_14	1	17,615	*Escherichia* phage PBECO 4 (NC_027364.1)	4.35	*Caudovirales; Myoviridae*
PYO2014_20	4	16,677	*Enterococcus* phage VD13 (NC_024212.1)	20.55	*Caudovirales; Siphoviridae*
PYO2014_31	50	227,129	*Escherichia* phage PBECO 4 (NC_027364.1)	54.417	*Caudovirales; Myoviridae*
PYO2014 collapsed bins. (Category 5)					
PYO2014_3.16.29	3	54,712	*Proteus* phage pPM_01 (NC_028812.1)	64.733	*Caudovirales; Siphoviridae; unclassified Siphoviridae*
PYO2014_5	25	193,706	*Enterobacteria* phage GEC-3S (NC_025425.1)	90.11	*Caudovirales; Myoviridae; Tevenvirinae; T4virus*
PYO2014_21	22	180,343	*Shigella* phage SHFML-11 (NC_030953.1)	88.2	*Caudovirales; Myoviridae; Tevenvirinae; T4virus*
PYO2014_25	3	78,290	*Enterobacteria* phage 285P (NC_015249.1)	76.539	*Caudovirales; Podoviridae; Autographivirinae; T7virus*
PYO2014 special cases, including bins classified as non-phages. (Category 6)					
PYO2014_6	26	75,778	Uncultured phage WW-nAnB strain 2 (NC_026612.1)	1.91	unknown
PYO2014_15	1	20,152	uncultured Mediterranean phage uvMED-CGF-C24-MedDCM-OCT-S28-C185 (AP013656.1)	0.69	unknown
PYO2014_22	5	57,117	*Pseudomonas* phage O4 (NC_031274.1)	1.78	*dsDNA viruses, no RNA stage*
PYO2014_24	26	89,259	*Cronobacter* phage vB CsaM GAP161 (NC_019398.1)	42.11	*Caudovirales; Myoviridae; Tevenvirinae*

^#^ Means “number of”. The 5th column reports the ANI of the reference genome towards the bin.

**Table 10:** Global mutation distances between samples.

Sample	PYO97	PYO2000	PYO2014
PYO97	0		
PYO2000	0.113 ± 0.0006	0	
PYO2014	0.132 ± 0.0008	0.138 ± 0.0009	0

## Supplementary Materials

The following are available online at www.mdpi.com/1999-4915/9/11/328/s1, Table S1: Percentages of PYO97 and PYO2014 reads mapping to MGmapper databases; Table S2: PYO97–Near-complete draft genome with high resemblance to reference phage, but no counterpart in PYO2014; Table S3: PYO2014–Near-complete draft genome with high resemblance to reference phage, but no counterpart in PYO97.
