# Supplementary material for: Metagenomic Analysis of Therapeutic PYO Phage Cocktails from 1997 to 2014
Source: Viruses. 2017 Nov 3;9(11):328. doi: 10.3390/v9110328 (PMC5707535; doi:10.3390/v9110328)
Supplement: Supplementary file 1 [file viruses-09-00328-s001.zip › PYO_supplementary/TableS1.docx]

**Table S1.** Percentages of PYO97 and PYO2014 reads mapping to MGmapper databases.

| Database | PYO97 % mapping reads | PYO2014 % mapping reads |
| --- | --- | --- |
| notPhiX | 100 | 100 |
| Bacteria | 0.034 | 0.290 |
| Archaea | 0 | 0 |
| MetaHitAssembly | 0.005 | 0 |
| HumanMicrobiome | 0.219 | 0 |
| Bacteria_draft | 0 | 0.001 |
| Human | 0.002 | 0 |
| Virus | 77.837 | 45.120 |
| Fungi | 0.007 | 0.001 |
| Unmapped | 21.895 | 54.588 |
