# Supplementary material for: Metagenomic Analysis of Therapeutic PYO Phage Cocktails from 1997 to 2014
Source: Viruses. 2017 Nov 3;9(11):328. doi: 10.3390/v9110328 (PMC5707535; doi:10.3390/v9110328)
Supplement: Supplementary file 1 [file viruses-09-00328-s001.zip › PYO_supplementary/TableS2.pdf]

Table S2

PYO97: Near-complete draft genome with high resemblance to reference phage and counterpart in PYO2014. (Category 1)

Phage draft genome	No. of contigs in draft genome	Total no. of basepairs (bp)	Closest match among 3889 reference phages from NCBI	Reference phage size (bp)	ANI (%) between phage draft genome and closest reference phage	Closest match among phage draft genomes in PYO2014	ANI (%) between phage draft genome in PYO97 (used as db) and closest match among phage draft genomes in PYO2014 (used as query)	Comment	Additional analysis			Predicted host by HostPhinder	Reliability score linked to host prediction
PYO97_4	1	149,561	Enterococcus phage EFDG1 (NC_029009.1)	147,589	89.77	PYO2014_27_42.21.133.90.116.20.14.109.73	90.0602		BLAST ATLAS of contigs from PYO97_4 and PYO2014_27_42.21.133.90.116.20.14.109.73 as outer rings against Enterococcus phage EFDG1 as reference	BLAST ATLAS of contigs against Enterococcus phage EFDG1 as reference		Enterococcus faecalis	6.80E-01
PYO97_10_85.139.47.48.59.38.5.35.15.78.55.44.14	13	344,749	Escherichia phage PBECO 4 (NC_027364.1)	348,113	90.848	PYO2014_31	83.8749	Escherichia phage 121Q is the best reference genome for PYO2014_31 and has 89.976% ANI towards this bin. The best common reference is Escherichia phage PBECO 4.	BLAST Atlas of bin vs Escherichia phage PBECO 4	BLAST Atlas of bin and PYO2014_31 with Escherichia phage PBECO 4 as reference	BLAST Atlas of contigs vs Escherichia phage PBECO 4	Escherichia coli	7.90E-01
PYO97_11	1	72,136	Pseudomonas phage PEV2 (NC_031063.1)	72,697	97.37	PYO2014_26.13	97.2065		BLAST ATLAS of PYO97_11 and PYO2014_26.13 against Pseudomonas phage PEV2 as reference.	BLAST ATLAS of contig against Pseudomonas phage PEV2 as reference		Pseudomonas aeruginosa	7.70E-01
PYO97_15	1	44,667	Pseudomonas phage TL (NC_023583.1)	45,696	92.02	PYO2014_12	91.222		BLAST ATLAS with Pseudomonas phage TL as the reference and PYO97_15 and PYO2014_12 as the outer rings			Pseudomonas aeruginosa	7.70E-01
PYO97_16	1	130,932	Staphylococcus phage Sb-1 (HQ163896.1)	127,188	96.86	PYO2014_30	94.077		BLAST ATLAS with Staphylococcus phage Sb-1 as the reference and PYO97_16 and PYO2014_30 as the outer rings			Staphylococcus aureus	6.70E-01
PYO97_29	1	169,029	Shigella phage SHFML-11 (NC_030953.1)	170,650	89.959	PYO2014_21	89.72	The best ref is Enterobacteria phage RB14 (NC_012638.1) 165429bp; 91.74% ANI.Shigella phage SHFML-11 is the best common reference between PYO97_29 and PYO2014_21.	BLASTAtlas PYO2014_21 and PYO97_29 with Shigella as reference	BLAST Atlas of PYO97_29 against Enterobacteria phage RB14	BLAST ATLAS of PYO97_29 as reference and PYO2014_21	Escherichia coli	6.00E-01

PYO97 - Near-complete draft genome with high resemblance to reference phage, but no counterpart in PYO2014 (Category 2)

Phage draft genome	No. of contigs in draft genome	Total no. of basepairs (bp)	Closest match among 3889 reference phages from NCBI	Reference phage size (bp)	ANI (%) between phage draft genome and closest reference phage	Closest match among phage draft genomes in PYO2014	ANI (%) between phage draft genome in PYO97 (used as db) and closest match among phage draft genomes in PYO2014 (used as query)	Comment	Additional analysis			Predicted host by HostPhinder	Reliability score linked to host prediction
PYO97_7	7	166,126	Klebsiella phage vB KpnM KpV477 (NC_031087.1)	168,272	88.66	PYO2014_21	34.04		BLAST Atlas of bin against Klebsiella phage vB KpnM KpV477	BLAST Atlas of PYO97_7, PYO2014_21 and PYO97_29 towards Shigella phage SHFML-11	Medium resemblance to phage in PYO2014. However 35.343 %ANI of Shigella phage SHFML-11, PYO2014_21 ref, towards PYO97_7	Klebsiella pneumoniae	6.70E-01
PYO97_8	1	38,419	Enterobacteria phage 285P (NC_015249.1)	39,270	79.568	PYO2014_25	66.3891	PYO2014_25 is classified as family bin (overlapping contigs). The best matching is Erwinia phage FE44 (NC_022744.1), 81.047% ANI . Erwinia is a genus of Enterobacteriaceae (likeE. coli), but mostly plant pathogenic! Enterobacteria phage 285P (NC_015249.1) is the closes shared reference between PYO97_8 and PYO2014_25 and ranks 3rd among the best matching references	BLASTAtlas of PYO97_8 and PYO2014_25 with Enterobacteria phage 285P as reference	BLAST ATLAS of contigs from PYO2014_25 against contig from PYO97_8 as reference	BLAST ATLAS of contigs against Erwinia phage FE44 as reference.	Escherichia coli	4.10E-01
PYO97_22.3.14	3	109,428	Salmonella phage Shivani (NC_028754.1)	120,098	95.33	PYO2014_7	13.49		BLAS Atlas of contigs against Salmonella ohage Shivani	BLAST Atlas of bin against Salmonella phage Shivani		Salmonella enterica	7.70E-01
PYO97_24	1	44,541	Proteus phage PM 85 (NC_027379.1)	43,642	92.726	PYO2014_11	0.533		BLAST ATLAS of contigs against Proteus phage PM 85 as reference			Proteus mirabilis	7.00E-01

PYO97_32	3	47,235	Salmonella phage vB SenS-Ent1 (HE775250.1)	42,391	86.967	PYO2014_24	0.134606	The fact that it's more than 10% larger than the reference is due to contigs overlapping	BLAST ATLAS of contigs against Salmonella phage vB SenS-Ent1 as reference	BLAST Atlas of bin against Salmonella phage vB SenS-Ent1		Salmonella enterica	4.30E-01

PYO97 - Partial draft genome with low/medium resemblance to reference phage and counterpart in PYO2014. (Category 3)

Phage draft genome	No. of contigs in draft genome	Total no. of basepairs (bp)	Closest match among 3889 reference phages from NCBI	Reference phage size (bp)	ANI (%) between phage draft genome and closest reference phage	Closest match among phage draft genomes in PYO2014	ANI (%) between phage draft genome in PYO97 (used as db) and closest match among phage draft genomes in PYO2014 (used as query)	Comment	Additional analysis			Predicted host by HostPhinder	Reliability score linked to host prediction
PYO97_27.21	2	37,487	Yersinia phage phi80-18 (NC_019911.1)	42,081	22.104	PYO2014_28	88.013		BLAST Atlas of PYO97_21 and PYO97_27 against common reference Yersinia phage phi80-18	Blast Atlas of PYO97_27.21 and PYO2014_28 towards Yersinia phage phi80-18	Blast Atlas of PYO2014_28 with PYO97_27.21 as reference	Yersinia enterocolitica	1.80E-03

PYO97 - Partial draft genome with no resemblance to reference phage and no counterpart in PYO2014. (Category 4)

Phage draft genome	No. of contigs in draft genome	Total no. of basepairs (bp)	Closest match among 3889 reference phages from NCBI	Reference phage size (bp)	ANI (%) between phage draft genome and closest reference phage	Closest match among phage draft genomes in PYO2014	ANI (%) between phage draft genome in PYO97 (used as db) and closest match among phage draft genomes in PYO2014 (used as query)	Comment	Additional analysis			Predicted host by HostPhinder	Reliability score linked to host prediction
PYO97_1	1	11,445	Escherichia phage vB EcoM AYO145A (NC_028825.1)	87,372	10.99	PYO2014_10	0.178		BLAST ATLAS of contig against Escherichia phage vB EcoM Alf5 as reference			Escherichia coli	4.70E-02
PYO97_5	3	29,155	Pseudomonas phage vB Pae-TbilisiM32 (JQ307386.1)	42,966	68.72	PYO2014_19	0.12		BLAST ATLAS of contigs against reference			Pseudomonas aeruginosa	5.60E-01
PYO97_9	1	10,727	Salmonella phage BP63 (NC_031250.1)	52,437	19.779	PYO2014_18	0.07		BLAST ATLAS of contig against Salmonella phage BP63 as reference			No_significant_match_found	
PYO97_10_3.8.10.28.42	5	343,801	Escherichia phage 121Q (NC_025447.1)	348,532	28.408	PYO2014_31	33.3208		BLAST Atlas of bin vs Escherichia phage 121Q	BLAST Atlas of bin and PYO2014_31 with Escherichia phage 121Q as reference	BLAST Atlas of contigs vs Escherichia phage 121Q	Cronobacter sakazakii	7.80E-03
PYO97_13	1	37,843	Hamiltonella virus APSE1 (NC_000935.1)	38,742	9.777	PYO2014_31	0.101339	Low/medium similarity to reference phage sequence.	BLAST ATLAS of contigs against reference			Hamiltonella defensa	1.90E-03
PYO97_17.30	7	90,209	Salmonella phage SSE121 (NC_027351.1)	147,745	58.832	PYO2014_18	3.633		BLAST Atlas of PYO97_30 and PYO97_17 against Salmonella phage SSE-121. Coverage of PYO97_17 from 12.757 to 21.05; coverage of PYO97_30 : 22.975			Salmonella enteritidis	5.40E-01
PYO97_20	1	90,712	Cronobacter phage vB CsaP GAP52 (NC_019402.1)	76,631	19.54	PYO2014_2	5.027	Low similarity to reference phage sequence. Low similarity to phage draft found in PYO2014	BLAST ATLAS of contigs against Escherichia phage KBNP1711 as reference.	BLAST ATLAS of PYO97_20 as reference and PYO2014_2		Cronobacter sakazakii	3.20E-03
PYO97_25	1	25,293	Proteus phage pPM_01 (NC_028812.1)	58,546	41.01	PYO2014_3.16.29	68.8522	PYO2014 --> ANI (%)<br>3 --> 84.378<br>16 --> 87.809<br>**29 --> 48.973**	BLAST ATLAS of PYO97_25 and PYO2014_16 as the outer rings and the reference as the inner ring	BLAST ATLAS of PYO97_25 against reference genome		Proteus mirabilis	2.80E-01
PYO97_26	5	171,908	Cronobacter phage S13 (NC_028773.1)	182,145	45.28	PYO2014_21	7.97	Medium resemblance to reference phage	BLAST ATLAS of bin against reference			Cronobacter sakazakii	9.00E-03
PYO97_28	5	30,952	Salmonella phage 21 (NC_029050.1)	51,896	21.43	PYO2014_18	0.83		BLAST ATLAS of contigs against reference			Salmonella enteritidis	1.10E-01
PYO97_31	3	69,885	Salmonella phage Felix 01 (NC_005282.1)	86,155	75.359	PYO2014_15	0.591	Almost 20,000 bp shorter than ref. sequence. Maybe partial.	BLAST ATLAS of contigs against Enterobacteria phage Felix 01 as reference			Escherichia coli	4.60E-01

PYO97 - Collapsed bins. (Category 5)

Phage draft genome	No. of contigs in draft genome	Total no. of basepairs (bp)	Closest match among 3889 reference phages from NCBI	Reference phage size (bp)	ANI (%) between phage draft genome and closest reference phage	Closest match among phage draft genomes in PYO2014	ANI (%) between phage draft genome in PYO97 (used as db) and closest match among phage draft genomes in PYO2014 (used as query)	Comment	Additional analysis			Predicted host by HostPhinder	Reliability score linked to host prediction

PYO97_12	5	55,452	Enterococcus phage IME-EFm5 (NC_028826.1)	42,265	69.288	PYO2014_1	93.401	The k-mer coverage values of overlapping contigs vary between 1.803 and 3.871. While the non overlapping contig has a k-mer coverage of 6.636	BLAST ATLAS of bin against Enterococcus phage IME-EFm5 as reference.	BLAST of contigs from PYO97_12 against PYO2014_1	PYO97_12 and PYO2014_1 look more similar to each other than each does to the reference Enterococcus phage IME-EFm5	Enterococcus faecium	2.00E-01	BLAST Atlas of contigs against reference	BLASTAtlas PYO2014_1 and PYO97_12 with Enterococcus phage IME-EFm5 as reference
PYO97_23	5	73,434	Enterococcus phage VD13 (NC_024212.1)	55,726	74.273	PYO2014_20	76.5524		BLAST ATLAS of contigs against Enterococcus phage VD13 as reference.	BLAST Atlas of bin against Enterococcus phage VD13	BLAST ATLAS of PYO2014_20 vs. PYO97_23	Enterococcus faecalis	2.70E-01		
PYO97 - Special cases, including bins classified as non-phages. (Category 6)															
Phage draft genome	No. of contigs in draft genome	Total no. of basepairs (bp)	Closest match among 3889 reference phages from NCBI	Reference phage size (bp)	ANI (%) between phage draft genome and closest reference phage	Closest match among phage draft genomes in PYO2014	ANI (%) between phage draft genome in PYO97 (used as db) and closest match among phage draft genomes in PYO2014 (used as query)	Comment	Additional analysis			Predicted host by HostPhinder	Reliability score linked to host prediction		
PYO97_2	1	11,313	uncultured Mediterranean phage uvMED-GF-C25-MedDCM-OCT-S33-C258 (AP014078.1)	6,279	0.704	PYO2014_14	0.24	Ref. phage sequence is partial	Does not resemble any other known phage sequence	The bin has 0.275% ANI towards phages in public databases. Possibly it is not a phage.		No_significant_match_found			
PYO97_6	8	23,397	uncultured Mediterranean phage uvMED-CGF-C14B-MedDCM-OCT-S36-C258 (AP013800.1)	7,156	1.426	PYO2014_6	6.28648	Ref. phage sequence is partial	Does not resemble any other known phage sequence	The bin has 0% ANI towards phages in public databases. Possibly it is not a phage.		Clostridium perfringens	1.00E-03		
PYO97_18	1	11,354	Pseudomonas phage PRR1 (NC_008294.1)	3,573	0.984	PYO2014_10	0.403	The reference phage has ss-RNA as genome!	BLAST ATLAS of contigs against Pseudomonas phage PRR1 as reference	The bin has 7.625% ANI towards phages in public databases and 23.684% coverage of Shigella sonnei Ss046 chromosome. Possibly it is not a phage.		Escherichia coli	3.00E-03		
PYO97_19	10	284,533	Staphylococcus phage Sb-1 (HQ163896.1)	127,188	18.88	PYO2014_30	19.03	Large size of phage draft genome suggests sequences from more phages have been wrongly binned	Separate calculating of ANI for the contigs to try and resolve if contigs have been wrongly binned	The bin has 0% ANI towards phages in public databases. Possibly it is not a phage.		Bacillus anthracis	1.50E-02		
PYO97_33	3	10,088	uncultured Mediterranean phage uvMED-CGF-C23-MedDCM-OCT-S24-C232 (AP013582.1)	5,75	1.131	PYO2014_14	0.786	Ref. phage sequence is partial	Does not resemble any other known phage sequence	The bin has 1.58794% ANI towards phages in public databases		Klebsiella pneumoniae	3.70E-04		
