# Supplementary material for: Metagenomic Analysis of Therapeutic PYO Phage Cocktails from 1997 to 2014
Source: Viruses. 2017 Nov 3;9(11):328. doi: 10.3390/v9110328 (PMC5707535; doi:10.3390/v9110328)
Supplement: Supplementary file 1 [file viruses-09-00328-s001.zip › PYO_supplementary/TableS3.pdf]

Table S3

PYO2014: Near-complete draft genome with high resemblance to reference phage and counterpart in PYO97. (Category 1)

Phage draft genome	No. of contigs in draft genome	Total no. of basepairs (bp)	Closest match among 3889 reference phages from NCBI	Reference phage size (bp)	ANI (%) between phage draft genome and closest reference phage	Closest match among phage draft genomes in PYO97	ANI (%) between phage draft genome in PYO2014 (used as db) and closest match among phage draft genomes in PYO97 (used as query)	Comment	Additional analysis			Predicted host by HostPhinder	Reliability score linked to host prediction
PYO2014_1	1	42,721	Enterococcus phage IME-EFm5 (NC_028826.1)	42,265	70.16	PYO97_12	87.65	Similar phage draft genome found in PYO97, although PYO97_12 is 55,452 bp. PYO97_12 made up of overlapping contigs	BLASTAtlas of PYO2014_1 against Enterococcus phage	BLASTAtlas PYO2014_1 and PYO97_12 with Enterococcus phage IME-EFm5 as reference		Enterococcus faecium	1.80E-01
PYO2014_12	1	47,209	Pseudomonas phage TL (NC_023583.1)	45,696	97.91	PYO97_15	94.45		BLASTAtlas of PYO2014_12 against Pseudomonas Phage TL	BLAST ATLAS with Pseudomonas phage TL as the reference and PYO97_15 and PYO2014_12 as the outer rings		Pseudomonas aeruginosa	8.60E-01
PYO2014_27_42. 21.133.90.116.20.14. 109.73	9	138,228	Enterococcus phage EFDG1 (NC_029009. 1)	147,589	81.548	PYO97_4	83.887		BLAST ATLAS of contigs PYO2014_27_42, PYO2014_27_21, PYO2014_27_133, PYO2014_27_90, PYO2014_27_116, PYO2014_27_20, PYO2014_27_14, PYO2014_27_109, PYO2014_27_73 against Enterococcus phage EFDG1	BLAST Atlas of bin against Enterococcus phage EFDG1 (NC_029009.1)		Enterococcus faecalis	5.50E-01
PYO2014_30	1	138,269	Staphylococcus phage ISP (FR852584.1)	138,339	99.36	PYO97_16	99.44		BLASTAtlas of PYO2014_30 against Staphylococcus phage ISP	BLAST ATLAS with Staphylococcus phage Sb-1 as the reference and PYO97_16 and PYO2014_30 as the outer rings		Staphylococcus aureus	7.00E-01

PYO2014 - Near-complete draft genome with high resemblance to reference phage, but no counterpart in PYO97. (Category 2)

Phage draft genome	No. of contigs in draft genome	Total no. of basepairs (bp)	Closest match among 3889 reference phages from NCBI	Reference phage size (bp)	ANI (%) between phage draft genome and closest reference phage	Closest match among phage draft genomes in PYO97	ANI (%) between phage draft genome in PYO2014 (used as db) and closest match among phage draft genomes in PYO97 (used as query)	Comment	Additional analysis			Predicted host by HostPhinder	Reliability score linked to host prediction
PYO2014_2	1	76,529	Escherichia phage ECBP2 (NC_018859. 1)	77,315	77.91	PYO97_20	4.2		BLAST ATLAS of contig with Escherichia phage ECBP2 as reference			Escherichia coli	2.50E-01
PYO2014_4	1	282,352	Pseudomonas phage phiKZ. (NC_004629. 1)	280,334	94.53	PYO97_33	0.68		BLAST ATLAS of bin with Pseudomonas phage phiKZ as reference			Pseudomonas aeruginosa	7.80E-01
PYO2014_8	1	36,807	Enterococcus phage EFAP-1 (NC_012419. 1)	21,115	74.45	PYO97_12	33.36	Significantly larger than reference genome. Partial genome similar to PYO97_12	BLAST Atlas of PYO2014_8 against Enterococcus phage EFAP-1	BLAST of PYO97_12 as reference and PYO2014_1 and PYO2014_8 as outer rings	BLAST Atlas of PYO2014_8 as reference and PYO97_12 and Enterococcus phage EFAP-1 as outer rings	Enterococcus faecalis	3.70E-01
PYO2014_17	1	88,099	Pseudomonas phage CHA P1 (NC_022974. 1)	88,255	94.91	PYO97_28	0.35		BLAST ATLAS of contig with Pseudomonas phage CHA P1 as reference			Pseudomonas aeruginosa	6.90E-01
PYO2014_18	1	147,760	Enterobacteria phage phi92 (NC_023693.1)	148,612	91.5	PYO97_17.30	5.92		BLAST ATLAS of PYO2014_18 and PYO97_30 against Enterobacteria phage phi92 as reference	BLAST Atlas of PYO2014_18 Enterobacteria phage phi92 as reference		Escherichia coli	7.30E-01
PYO2014_23	1	38,847	Enterobacteria phage K1F (NC_007456.1)	39,704	82.27	PYO97_8	25.52		BLAST ATLAS of contig against reference.			Escherichia coli	4.20E-01

PYO2014_26.13	2	65,818	Pseudomonas phage PEV2 (NC_031063.1)	72,697	90.705	PYO97_11	69.978	Partial genome with corresponding bin in PYO97	BLASTAtlas PYO2014_26.13 with Pseudomonas phage PEV2 as reference	PYO2014_26 and PYO2014_13 against Pseudomonas phage PEV2	BLASTAtlas PYO97_11 and PYO2014_26.13 with Pseudomonas phage PEV2 as reference	Pseudomonas aeruginosa	8.60E-01
PYO2014_27_16.5	2	139,828	Enterococcus phage EFLK1 (NC_029026.1)	130,952	90.812	PYO97_4	35.735		BLAST ATLAS of contigs PYO2014_27_16 and PYO2014_27_5 against Enterococcus phage EFLK1	Contigs of the original bin with Enterococcus phage EFLK1 as reference		Enterococcus faecalis	7.20E-01
PYO2014 - Partial draft genome with low/medium resemblance to reference phage and counterpart in PYO97. (Category 3)													
Phage draft genome	No. of contigs in draft genome	Total no. of basepairs (bp)	Closest match among 3889 reference phages from NCBI	Reference phage size (bp)	ANI (%) between phage draft genome and closest reference phage	Closest match among phage draft genomes in PYO97	ANI (%) between phage draft genome in PYO2014 (used as db) and closest match among phage draft genomes in PYO97 (used as query)	Comment	Additional analysis			Predicted host by HostPhinder	Reliability score linked to host prediction
PYO2014_28	1	33,115	Yersinia phage phi80-18 (NC_019911.1)	42,081	16.79	PYO97_27.21	77.7455		BLAST ATLAS of PYO2014_28 with Yersinia phage phi80-18 as reference	Blast Atlas of PYO97_27.21 and PYO2014_28 towards Yersinia phage phi80-18	Blast Atlas of PYO97_27.21 with PYO2014_28 as reference	Yersinia enterocolitica	1.50E-03
PYO2014 - Partial draft genome with no resemblance to reference phage and no counterpart in PYO97. (Category 4)													
Phage draft genome	No. of contigs in draft genome	Total no. of basepairs (bp)	Closest match among 3889 reference phages from NCBI	Reference phage size (bp)	ANI (%) between phage draft genome and closest reference phage	Closest match among phage draft genomes in PYO97	ANI (%) between phage draft genome in PYO2014 (used as db) and closest match among phage draft genomes in PYO97 (used as query)	Comment	Additional analysis			Predicted host by HostPhinder	Reliability score linked to host prediction
PYO2014_7	1	103,078	Escherichia phage bV EcoS AKFV33 (HQ665011.1)	108,853	14.32	PYO97_22.3.14	12.8684		BLAST Atlas of bin against ref			Escherichia coli	2.60E-03
PYO2014_9	1	37,468	Enterococcus phage vB IME197 (NC_028671.1)	41,307	15.18	PYO97_12	0.57		BLAST ATLAS of contigs against reference			Enterococcus faecalis	3.20E-03
PYO2014_19	1	43,272	Pseudomonas phage vB PaeP Tr60 Ab31 (NC_023575.1)	45,550	45.35	PYO97_5	0.33		BLAST ATLAS of contig against reference			Pseudomonas aeruginosa	6.10E-02
PYO2014_10	1	10,736	Escherichia phage PBECO 4 (NC_027364.1)	348,113	3.06	PYO97_10_85.139.47.48.59.38.5.35.15.78.55.44.14	3.05757		BLAST ATLAS of contig with Escherichia phage PBECO 4 as reference			Escherichia coli	1.10E-02
PYO2014_11	1	13,190	Escherichia phage PE3-1 (NC_024379.1)	39,093	29.52	PYO97_8	9.23	Might belong to same family as 2014_23, 97_8, and 2014_25	BLAST ATLAS of contig with Escherichia hage PE3-1 as reference			Escherichia coli	1.20E-01
PYO2014_14	1	17,615	Escherichia phage PBECO 4 (NC_027364.1)	348,113	4.35	PYO97_33	3.08		BLAST ATLAS of contig with Escherichia phage PBECO 4 as reference			Escherichia coli	2.10E-02
PYO2014_20	4	16,677	Enterococcus phage VD13 (NC_024212.1)	55,726	20.55	PYO97_23	24.99		BLAST Atlas of bin towards Enterococcus phage VD13	BLASTAtlas of PYO014_20 and PYO97_23 with Enterococcus phage VD13 as reference		Enterococcus faecalis	9.80E-02
PYO2014_31	50	227,129	Escherichia phage PBECO 4 (NC_027364.1)	348,113	54.417	PYO97_10_85.139.47.48.59.38.5.35.15.78.55.44.14.fa	55.4234		BLASTAtlas of PYO97_10_85.139.47.48.59.38.5.35.15.78.55.44.14 and PYO2014_31 with Escherichia phage PBECO 4 as reference	BLAST ATLAS of bin with Escherichia phage 121Q as reference	Best reference is Escherichia phage 121Q (NC_025447.1), which has 54.72% ANI. Escherichia pahge PBECO 4 is the best common reference between PYO2014_31 and its counterpart in PYO97.	Escherichia coli	2.00E-01
PYO2014 - Collapsed bins. (Category 5)													

Phage draft genome	No. of contigs in draft genome	Total no. of basepairs (bp)	Closest match among 3889 reference phages from NCBI	Reference phage size (bp)	ANI (%) between phage draft genome and closest reference phage	Closest match among phage draft genomes in PYO2014	ANI (%) between phage draft genome in PYO97 (used as db) and closest match among phage draft genomes in PYO2014 (used as query)	Comment	Additional analysis			Predicted host by HostPhinder	Reliability score linked to host prediction
PYO2014_3.16.29	3	54,712	Proteus phage pPM_01 (NC_028812.1)	58,546	64.733	PYO97_25	98.203	kmer-coverage 557.677 (121.679) Overlapping contigs, i.e. PYO2014_3 and PYO2014_16 have k-mer coverage 449.796 495.515 respectively while the unique contig, PYO2014_29, has k-mer coverage=727.721	BLAST ATLAS of PYO2014_3, 2014_29, and 2014_16 as outer rings against reference			Proteus mirabilis	5.80E-01
PYO2014_5	25	193,706	Enterobacteria phage GEC-3S (NC_025425.1)	163,424	90.11	PYO97_29	13.8		BLAST ATLAS of contigs against reference	BLAST Atlas of sorted contigs		Escherichia coli	7.80E-01
PYO2014_21	22	180,343	Shigella phage SHFML-11 (NC_030953.1)	170,650	88.2	PYO97_29	84.5	PYO97_29 has Enterobacteria phage RB14 (NC_012638.1) as reference, which has 84.406% ANI towards PYO2014_21. The common reference with the highest ANI is Shigella phage SHFML-11.	BLASTAtlas of PYO2014_21 against Shigella phage SHFML-11	BLASTAtlas PYO2014_21 and PYO97_29 with Shigella as reference	BLASTAtlas of contigs against Shigella phage SHFML-11	Shigella sonnei	8.40E-01
PYO2014_25	3	78,290	Enterobacteria phage 285P (NC_015249.1)	39,270	76.539	PYO97_8	76.16		PYO2014_25 contigs agianst reference	PYO97_8 and PYO2014_25 against reference		Escherichia coli	4.10E-01
PYO2014 - Special cases, including bins classified as non-phages. (Category 6)													
Phage draft genome	No. of contigs in draft genome	Total no. of basepairs (bp)	Closest match among 3889 reference phages from NCBI	Reference phage size (bp)	ANI (%) between phage draft genome and closest reference phage	Closest match among phage draft genomes in PYO2014	ANI (%) between phage draft genome in PYO97 (used as db) and closest match among phage draft genomes in PYO2014 (used as query)	Comment	Additional analysis			Predicted host by HostPhinder	Reliability score linked to host prediction
PYO2014_6	26	75,778	Uncultured phage WW-nAnB strain 2 (NC_026612.1)	5,077	1.91	PYO97_6	10.51	Ref. phage is partial Longest contig 5513 and shorter 2013	Test with MetaPhinder if it is a phage	The bin has 1.862% ANI towards phages in public databases. Possibly it is not a phage.		Clostridium perfringens	3.10E-03
PYO2014_15	1	20,152	uncultured Mediterranean phage uvMED-CGF-C24-MedDCM-OCT-S28-C185 (AP013656.1)	5,986	0.69	PYO97_21	0.2		Test with MetaPhinder if it is a phage	The bin has 5.226% ANI towards phages in public databases. Possibly it is not a phage.		Pseudomonas syringae	2.50E-04
PYO2014_22	5	57,117	Pseudomonas phage O4 (NC_031274.1)	50,509	1.78	PYO97_33	0.28		BLAST ATLAS of contigs with Pseudomonas phage O4 as reference	The bin has 8.52427% ANI towards phages in public databases. Possibly it is not a phage.		Vibrio parahaemolyticus	3.10E-04
PYO2014_24	26	89,259	Cronobacter phage vB CsaM GAP161 (NC_019398.1)	178,193	42.11	PYO97_7	3.79	Shorter contig 2014 and longest 6898	BLAST ATLAS of contigs with Cronobacter phage vB CsaM GAP161 as reference	79.4769% ANI towards phages in public databases. Short contigs.		Cronobacter sakazakii	1.90E-01
